# West Syndrome in South Iran: Electro-Clinical Manifestations

**Published:** 2013

**Authors:** Ali Akbar ASADI-POOYA, Mohaddese SHARIFZADE

**Affiliations:** 1 Neurosciences Research Center, Shiraz University of Medical Sciences, Shiraz, Iran; 2 2.Jefferson Comprehensive Epilepsy Center, Department of Neurology, Thomas Jefferson University, Philadelphia, USA

**Keywords:** Electroencephalography, Spasms, West syndrome

## Abstract

**Objective:**

We aimed to determine the clinical and electroencephalographic (EEG) characteristics of the patients with West syndrome (WS) in south Iran.

**Materials & Methods:**

In this retrospective study, all patients with a clinical diagnosis of WS were recruited in the outpatient epilepsy clinic at Shiraz University of Medical Sciences between September 2008 and May 2012. Age, gender, age at seizure onset, seizure type(s), epilepsy risk factors, EEG and imaging studies of all patients were registered routinely.

**Results:**

During the study period, 2500 patients with epilepsy were registered at our epilepsy clinic. Thirty-two patients (1.3%) were diagnosed to have WS. Age of onset (mean ± standard deviation) was 4.99 ± 3.06 months. Sixteen patients were male and 16 were female. Nine (28.1%) were reported to have two or more seizure types and 23 (71.8%) had one seizure type (epileptic spasms). At referral, no developmental delay was detected in two patients and in the rest, a mild to severe delay was noted. Electroencephalography showed typical hypsarrhythmia in 59.4% of our patients and modified hypsarrhythmia or atypical presentations were seen in 40.6%. Two patients had pyridoxine (B6)-dependent seizures, confirmed by oral B6 trial.

**Conclusion:**

Variants of the classical triad of WS including other seizure types, atypical EEG findings, and normal psychomotor function at the beginning could be observed in some patients. Rarely, treatable genetic disorders (e.g., pyridoxine-dependent seizures) should be considered in those in whom no other diagnosis is evident.

## Introduction

West syndrome (WS) is characterized by epileptic spasms (formerly, infantile spasms), a specific electroencephalographic (EEG) pattern (hypsarrhythmia), and psychomotor retardation beginning in the first year of life (1-5). This classic triad may vary in age (with its onset ranging from neonatal period to four years), spasms (asymmetrical or combined with other seizure types), modified hypsarrhythmia, and psychomotor function which can be normal or delayed [5]. In the current study, we tried to determine the clinical and electroencephalographic characteristics of patients with WS in south Iran.

## Materials & Methods

In this cross-sectional retrospective chart review study, all patients with a clinical diagnosis of WS were recruited at the outpatient epilepsy clinic at Shiraz University of Medical Sciences, the only epilepsy clinic in south Iran, from September 2008 to May 2012. The diagnosis of WS was made based on the clinical and EEG grounds. All patients were visited by an epileptologist at our institution. Out-patient EEG (either awake or sleep) was performed in all patients at the time of referral. The high frequency filter was set to 70 hertz (Hz) and the low filter was at 0.16 Hz. In-patient video-EEG monitoring study was performed when considered necessary for confirmation of the diagnosis or planning the treatment strategy. The study time for the video-EEG monitoring was 120 minutes and recording both wakefulness and sleep was required in all patients. Brain imaging study (magnetic resonance imaging [MRI]) was performed when possible. Other tests (e.g., blood works) were requested based on the clinical judgment.

We studied the demographic, clinical, EEG, and imaging findings. Age, gender, age at seizure onset, seizure type(s), epilepsy risk factors (including history of perinatal complications or significant head trauma, history of febrile seizure, positive family history of epilepsy and parental consanguinity), EEG and imaging studies of all patients were recorded. Demographic variables and relevant clinical and EEG variables were descriptively summarized to characterize the study population. This study was approved by Shiraz University of Medical Sciences Review Board.

## Results

**Fig 1:**
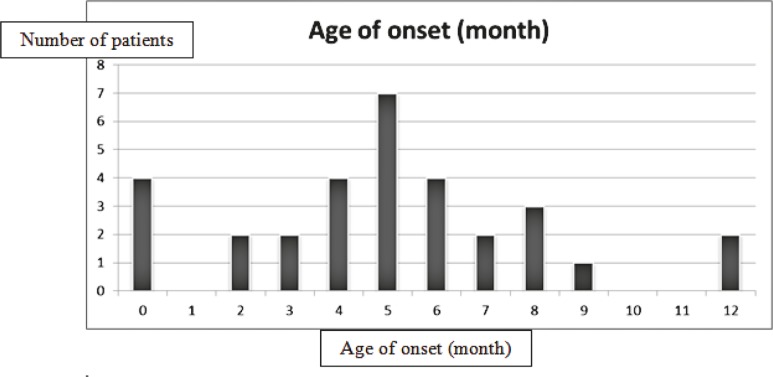
Age of onset in patients with West syndrome (mean ± standard deviation was 4.99 ± 3.06 months). Minimum age of onset was the neonatal period and maximum age was 12 months

During the study period, 2500 patients with epilepsy were registered at our epilepsy clinic. Thirty-two patients (1.3%) were diagnosed to have WS. Age of onset (mean± standard deviation) was 4.99 ± 3.06 months (range; neonatal period to 12 months of age). Figure 1 shows the age of onset in patients with WS.

Sixteen patients were male (50%) and 16 (50%) were male. Nine patients (28.1%) were reported to have two or more seizure types and 23 (71.8%) had only epileptic spasms. Epileptic spasms, tonic, atonic, generalized tonic-clonic, and myoclon seizures were reported in 30 (93.7%), 8 (25%), 2 (6.2%), 1 (3.1%), and 1 (3.1%) patients, respectively.

Epilepsy risk factors were reported to be as follow: perinatal complications in 12 patients (37.5%; including hypoxic-ischemic insults in 8 [25%] and sepsis, low- birth-weight, and hyperbilirubinemia in other four), family history of epilepsy in four (12.5%), and parental consanguinity in 13 (40.6%).

At referral, two patients did not have developmental delay while the rest had a mild to severe developmental delay. Many patients had medical or neurological comorbidities including cerebral palsy in 13 (40.6%), tuberous sclerosis in two (6.2%), intra-uterine infection by cytomegalovirus in one (3.1%), hypothyroidism in one (3.1%), and congenital heart disease in one (3.1%). We requested EEG for all patients at the time of referral (Table 1). Brain MRI was done only in 30 patients because other two could not afford its cost. The results are summarized in Table 2. Blood works were not performed in 10 patients. When it was performed, it was not conclusive in most cases. However, lactic acidosis and hyperammonemia were detected in four and two cases, respectively. Two other patients had pyridoxine (B6)-dependent seizures confirmed by oral B6 trial. Eight cases had new-onset disease diagnosed at our clinic and the rest were referred from other centers after being treated for some time. All patients with newly diagnosed WS had epileptic spasms. One also had tonic seizures. Five patients had hypsarrhythmia in their EEGs while three had multifocal spikes and slow spike-wave complexes. Brain MRI was normal in three patients but showed atrophy in two and schizencephaly in one.

## Discussion

West syndrome is a rare epileptic encephalopathy characterized by epileptic spasms, typical electroencephalographic patterns, and psychomotor retardation (1-5). The frequency of this syndrome was 1.3% in both children and adults in our clinic. This is similar to the previous reports in this regard (6, 7). In a study from Hong Kong, 1.9% of the children with epilepsy had WS (6). Clinical manifestations of WS are more or less similar in various studies. Sex ratio (male to female) and age of onset of WS in our study were similar to the previous reports, as well (8). The hallmark of this syndrome is epileptic spasms, as the prominent seizure type. In our study, all patients with new-onset WS and most treated patients (92%) had epileptic spasms, while other seizure types were reported in some patients, particularly in those who had the syndrome for some time and were being treated. In previous studies, similar results have been reported (7). Epilepsy risk factors particularly perinatal complications and comorbid neurological and medical conditions observed in our patients were also more or less similar to the previous studies (7). Electroencephalography showed typical hypsarrhythmia in 59.4% and modified hypsarrhythmia or atypical presentations in 40.6% of our patients. In a Taiwanese study, the rate of typical hypsarrhythmia was 46% (8). Atypical EEG presentations have been reported in previous studies (7) and EEG findings other than hypsarrhythmia should be expected in patients with WS. In our WS patients, brain imaging abnormalities were similar to the previous studies (7) showing normal results in about two-fifths and non-specific atrophy in almost one-thirds of the patients. Other laboratory studies are often normal or non-specific; however, in a minority of the patients, inborn errors of metabolism can be detected and at times, treated.

West syndrome is often considered as a drug-resistant disease. Although they are rarely treatable, genetic disorders should be considered in those in whom no other diagnosis is evident. Pyridoxine (B6)-dependent seizure is a rare autosomal recessive disorder that usually presents with intractable seizures including spasms in early years of life. The seizures can be completely controlled by administration of vitamin B6; however, if not treated promptly, irreversible neurological damage may occur. Vitamin B6 should be administered under EEG monitoring as a diagnostic test in all infants and young children with epilepsy when no other diagnosis is evident. Parenteral B6 injection confirms the diagnosis. Clinical seizures stop within a few minutes and epileptic EEG discharges subside within a few hours after the intravenous injection of 50 to 200 mg of B6 (9-11). Acute hypotonia and apnea has been reported after intravenous B6 administration to infants after long-time seizures. Resuscitation equipments should therefore be available during an intravenous trial (9). Alternatively, the disorder may be diagnosed by giving 15 mg/kg/ day of oral B6 to an infant who experiences frequent seizures. Complete control of the seizures within a week or so is confirmatory in these situations (9, 12). Once the diagnosis is confirmed, maintenance therapy (25 to 200 mg/day) should be indefinitely continued. Doses should be increased with advancing age or when intercurrent illnesses occur (9). Women with a child with vitamin B6-dependent seizures are recommended to receive this vitamin during subsequent pregnancies (9).

Since this study was a clinic-based series, it might not represent the full spectrum of patients with WS. Besides, many patients were referred to us after being treated for a while and their initial clinical and EEG findings could not be ascertained. Therefore, we did not try to classify the patients to idiopathic or symptomatic in this paper. 

**Table 1:** EEG Findings in Patients with West Syndrome

**Finding**	**All patients**	**New-onset patients**
	**Number (Percent)**	**Number (Percent)**
Background abnormality	25 (78.1)	8 (100)
Hypsarrhythmia	19 (59.4)	5 (62.5)
GPFA	10 (31.3)	2 (25)
Slow Spike-wave complex	8 (25)	3 (37.5)
Multifocal spikes	6 (18.8)	1 (12.5)
Burst-Suppression	1 (3.1)	0

GPFA: generalized paroxysmal fast activity.

**Table 2:** Brain MRI Findings in Patients with West Syndrome

**Finding**	**Number**	**Percent**
Normal	15	47
Brain atrophy	11	34.5
Hypoxic-ischemic changes	3	9
Schizencephaly	1	3
Not performed	2	6.5

**In conclusion, **WS is a rare age-dependent epileptic encephalopathy characterized by epileptic spasms, a specific EEG pattern (hypsarrhythmia), and psychomotor retardation which begins in the first year of life. It is often refractory to usual antiepileptic drugs. However, rarely, treatable genetic disorders (e.g., pyridoxine- dependent seizures) should be considered in those with no other evident diagnosis. The accurate determination of etiology is now becoming more possible (13).
